# An evaluation of the Technicon DPA*1 specific protein analyser

**DOI:** 10.1155/S146392469000027X

**Published:** 1990

**Authors:** M. J. Waterson, L. Skingle, C. P. Price

**Affiliations:** 1 Department of Clinical Biochemistry The London Hospital Whitechapel London E1 1BB UK; 2 Department of Clinical Chemistry Barking Hospital Upney Lane Essex Barking IGll 9LX UK


Journal of Automatic Chemistry, Vol. 12, No. 5 (September–October 1990), pp. 205–212

### An evaluation of the Technicon DPA*1 specific protein analyser

**M. J. Waterson†, L. Skingle and C. P. Price**

*Department of Clinical Biochemistry, The London Hospital, Whitechapel, London E1 1BB, UK*

*The DPA*1 analyser, a fully automated, discrete, random-access specific protein analyser has been evaluated. Eight, out of a current menu of 17 analytes, were assessed for precision, accuracy and calibration stability. Within-batch precision was excellent, generally being between 1% and 3%; between-batch precision was also good (3–6%). Method comparisons showed good agreement; the best correlations being obtained when the comparison methodology was also nephelometric.*

*Calibration stability was a minimum of one month in each case and there was no evidence of sample carry-over. The instrument was easy to use and offered a realistic analysis rate of approximately 100 completed tests per hour.*

Automated immunoassay of proteins in serum can be performed by means of turbidimetry or nephelometry [1]. The DPA*1 analyser is a fully automated, discrete random access specific protein analyser with measurement by rate nephelometry [2]. Although concentrations are determined by peak rate nephelometry, the system can kinetically monitor the antigen–antibody reaction of many tests simultaneously. The system incorporates sophisticated antigen excess checking procedures and automatic redilution of samples falling outside the analytical range of an assay.

The analytical performance of the DPA*1 was assessed for eight different analytes out of a current repertoire of 17. The aspects evaluated included precision, linearity, calibration stability, relative accuracy, carry-over, throughput and the validity of the antigen excess checking procedures.

### Materials and methods

### *The instrument*

The Technicon DPA*1 (supplied by Technicon UK, Basingstoke, Hampshire, UK) is a bench-top unit, comprising an analytical unit with built-in keyboard, a separate VDU and keyboard, and a printer. A single 13 amp power supply is required. Up to 12 analytes may be measured at any one time, the antiserum reagent being supplied, ready-to-use in bar-coded vials, each sufficient for 100 analyses. A single probe, with level detector, performs all liquid handling and mixing functions. A sample is initially diluted in 3·4% polyethylene glycol (PEG) causing the precipitation of any serum components that might interfere in the subsequent measurement. An aliquot of this diluted sample is mixed with antiserum and further PEG; the reaction is then monitored every 10 s for a period of 7 min. Measurement is by nephelometry using a 600 nm pulsed LED light source and a detection angle of 30°. Results are produced at the rate of three tests/min, irrespective of the number of samples or tests/sample. A single point calibrator is used, the standard curve parameters being encoded on the reagent bar-code label. Samples above and below the working range of the assay are automatically rediluted and reanalysed.

There are three methods of checking for antigen excess. Firstly, the time course of the reaction is automatically monitored, checking the length of lag phase and time to peak rate. Secondly, a second aliquot of sample can be added to the reaction mixture 7 min after completion of analysis – if no further reaction occurs the system is in antigen excess. Thirdly, if a rediluted sample is still below the lower limit of the assay, this could be due to a very low sample concentration; or to the presence of extreme antigen excess. To distinguish between the two, an aliquot of control sample is added to the reaction mixture – if no reaction takes place extreme antigen excess is indicated.

### Analytical methods and reagents

The instrument was used in accordance with the manufacturer's instructions; all calibrators and antiserum being supplied by Technicon UK. The comparison methodologies are shown in table 1, as are the sample volumes used by the DPA*1. Other standards and controls were obtained from Atlantic Antibodies (Incstar,

*Table 1. Method evaluation on the DPA*1.*

| Analyte | Sample volume (μl) | Comparison method |
|---|---|---|
| Immunoglobulin A | 16 | Beckman Array† |
| Immunoglobulin G | 10 | Beckman Array† |
| Immunoglobulin M | 25 | Beckman Array† |
| Alpha-1-antitrypsin | 15 | IL Monarch (Atlantic Antibodies antiserum)‡ |
| Transferrin | 10 | IL Multistat (Atlantic Antibodies antiserum) |
| Haptoglobin | 12 | IL Monarch (Atlantic Antibodies antiserum)‡ |
| Apolipoprotein A1 | 15 | IL Multistat (Orion kit) |
| Apolipoprotein B | 10 | IL Multistat (Immuno Kit) |

† Assayed in the Department of Immunology, The London Hospital.
‡ Assayed in the Department of Clinical Biochemistry, Addenbrookes Hospital, Cambridge.

†*Current address: Department of Clinical Chemistry, Barking Hospital, Upney Lane, Barking, Essex IG11 9LX, UK.*

0142–0453/90 $3.00 © 1990 Taylor & Francis Ltd.

Wokingham, UK), BCL (Lewes, UK), Behring (Hoescht) (Middlesex, UK), and the Protein Reference Unit (Sheffield, UK). External quality assessment samples (EQAS), with results, were supplied by the Department of Immunology, East Birmingham Hospital (Birmingham, UK).

*Precision*

Within-batch precision was assessed by analysing three pools of serum at high, medium and low concentrations. The pools were arranged randomly on the 45 place sample tray and random cups were assayed in duplicate to give a total of 20 determinations at each level from the 45 samples.

Between-day precision was assayed using a mixture of frozen pools and commercial quality-control material. The study covered a single calibration period for alpha-1-antitrypsin and the apolipoproteins, but more than one period for the other analytes.

*Calibration stability*

Each assay was calibrated on day 0, no further calibration being performed for a period of at least 35 days. The calibrator and a series of pools covering a wide concentration range were assayed as samples during the period to assess the stability of calibration.

*Linearity*

To determine the quality of the calibration produced using a single point calibrator a series of calibrators, in saline, of a high standard (SPS-02, Protein Reference Unit, Sheffield, UK) were made. The samples were assayed two weeks after a single point calibration.

*Accuracy*

At least 60 patient samples were assayed by the DPA*1 and by the comparison methodologies shown in table 1. In addition, a series of commercial calibrators and quality materials with quoted values and external quality assurance samples were analysed by the DPA*1.

*Carry-over*

Carry-over was estimated by running four sequences of a high pool (×3) and a low pool (×3) for immunoglobulin G.

*Throughput*

The time taken from commencement of the run to the final result printout was measured for a variety of workload patterns.

*Antigen excess checking*

A total of 31 known myeloma samples (15 IgG, 7 IgA and 9 IgM) were used to test the antigen excess software, consisting of both very high and suppressed immunoglobulin concentrations.

### Results

Within-batch and between-batch precision data are shown in tables 2 and 3, respectively. The results for within-batch precision show excellent performance in all cases – the majority of the coefficients of variation being well below 3%. Inter-assay precision was good, generally

*Table 2. Within-batch precision for eight analytes on the DPA*1 (N = 20 for each sample).*

| Analyte | Mean (g/l) | SD | CV (%) |
|---|---|---|---|
| Immunoglobulin A | 0·83 | 0·011 | 1·3 |
| | 1·82 | 0·018 | 1·0 |
| | 3·75 | 0·055 | 1·5 |
| Immunoglobulin G | 4·87 | 0·055 | 1·1 |
| | 10·6 | 0·10 | 1·0 |
| | 28·7 | 0·59 | 2·1 |
| Immunoglobulin M | 0·54 | 0·0087 | 1·6 |
| | 1·28 | 0·044 | 3·4 |
| | 1·87 | 0·068 | 3·6 |
| Alpha-1-antitrypsin | 0·97 | 0·0130 | 1·3 |
| | 1·99 | 0·034 | 1·7 |
| | 3 33 | 0·040 | 1·2 |
| Transferrin | 0·95 | 0·0138 | 1·4 |
| | 2·99 | 0·059 | 2·0 |
| | 5·32 | 0·158 | 3·0 |
| Haptoglobin | 0·81 | 0·0116 | 1·4 |
| | 1·85 | 0·032 | 1·7 |
| | 2·77 | 0·055 | 2·0 |
| Apolipoprotein A1 | 0·69 | 0·0131 | 1·9 |
| | 1·52 | 0·048 | 3·2 |
| | 1·96 | 0·013 | 2·1 |
| Apolipoprotein B | 0·53 | 0·011 | 2·1 |
| | 1·13 | 0·027 | 2·4 |
| | 1·57 | 0·021 | 1·4 |

*Table 3. Between-day precision for eight analytes on the DPA*1.*

| Analyte | Number | Mean | SD | CV (%) |
|---|---|---|---|---|
| Immunoglobulin A | 19 | 0·845 | 0·021 | 2·5 |
| | 20 | 2·15 | 0·067 | 3·1 |
| | 20 | 4·38 | 0·187 | 4·3 |
| Immunoglobulin G | 19 | 4·23 | 0·120 | 2·8 |
| | 20 | 10·85 | 0·576 | 5·3 |
| | 20 | 24·40 | 1·410 | 5·8 |
| Immunoglobulin M | 20 | 0·48 | 0·023 | 4·8 |
| | 19 | 1·17 | 0·049 | 4·2 |
| | 20 | 2·95 | 0·104 | 4·8 |
| Alpha-1-antitrypsin | 18 | 0·74 | 0·035 | 4·7 |
| | 20 | 1·94 | 0·067 | 3·5 |
| | 19 | 3·48 | 0·104 | 3·0 |
| Transferrin | 19 | 1·18 | 0·740 | 4·7 |
| | 20 | 2·25 | 0·151 | 6·7 |
| | 20 | 5·28 | 0·299 | 5·7 |
| Haptoglobin | 20 | 0·59 | 0·019 | 3·1 |
| | 20 | 1·25 | 0·063 | 5·0 |
| | 18 | 2·23 | 0·131 | 5·9 |
| Apolipoprotein A1 | 20 | 0·82 | 0·051 | 6·1 |
| | 20 | 1·19 | 0·073 | 6·2 |
| | 20 | 1·47 | 0·082 | 5·6 |
| Apolipoprotein B | 20 | 0·52 | 0·027 | 5·2 |
| | 20 | 0·77 | 0·033 | 4·0 |
| | 20 | 1·28 | 0·051 | 6·3 |

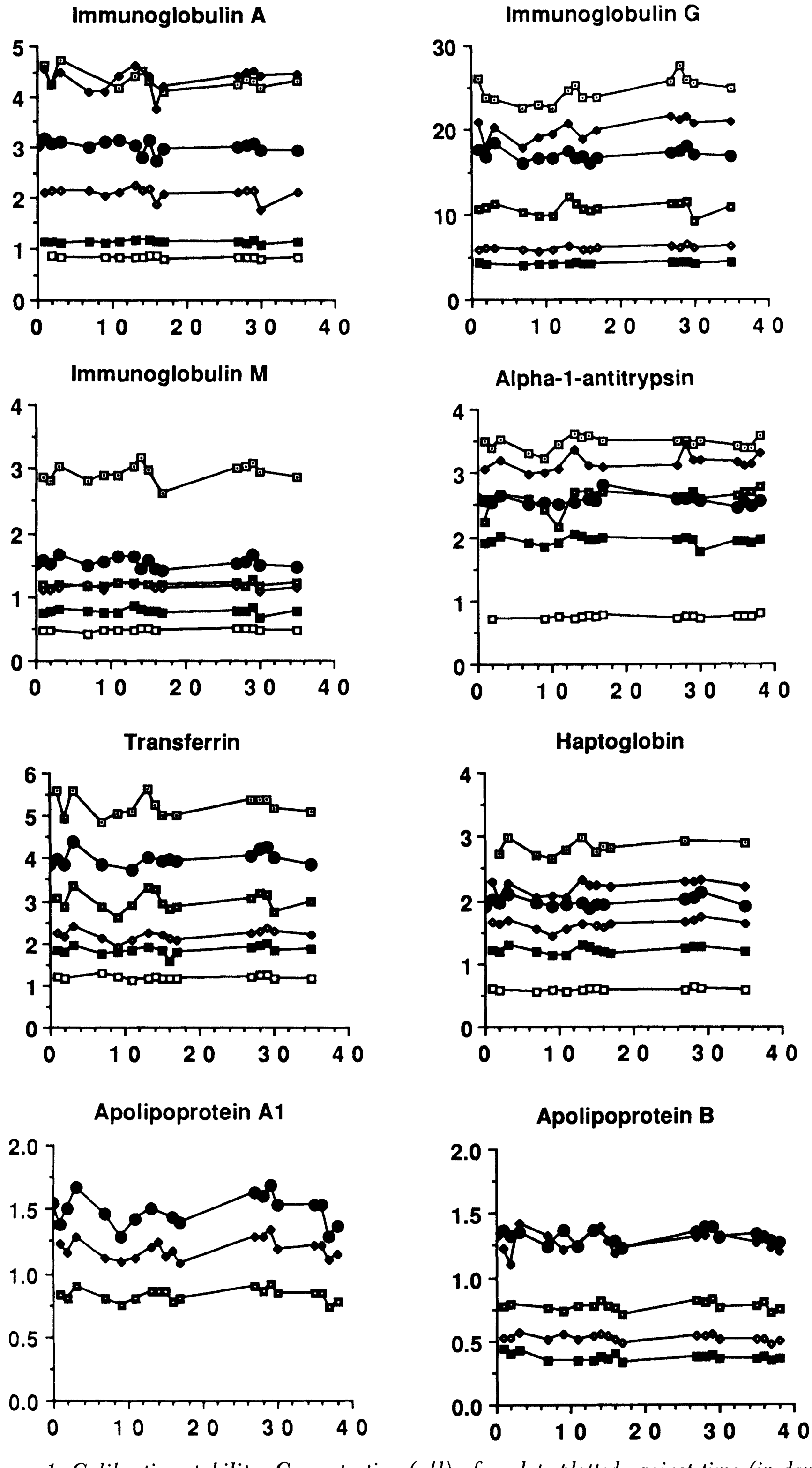

*Figure 1. Calibration stability. Concentration (g/l) of analyte plotted against time (in days).*

being between 3% and 5%. This probably could be improved upon in routine use as the data included a short time of poor precision, which was probably caused by a slightly blocked probe.

### *Calibration stability*

The measured concentrations of various pools of the different analytes over a minimum of 35 days are shown in figure 1. In all cases, calibration appeared stable for this period of time, i.e. greater than the 2-week period claimed by the suppliers.

### *Linearity*

All methods were linear over the range of the assay, as shown in figure 2, confirming the suitability of the curve-fit parameters encoded on the reagent bar code.

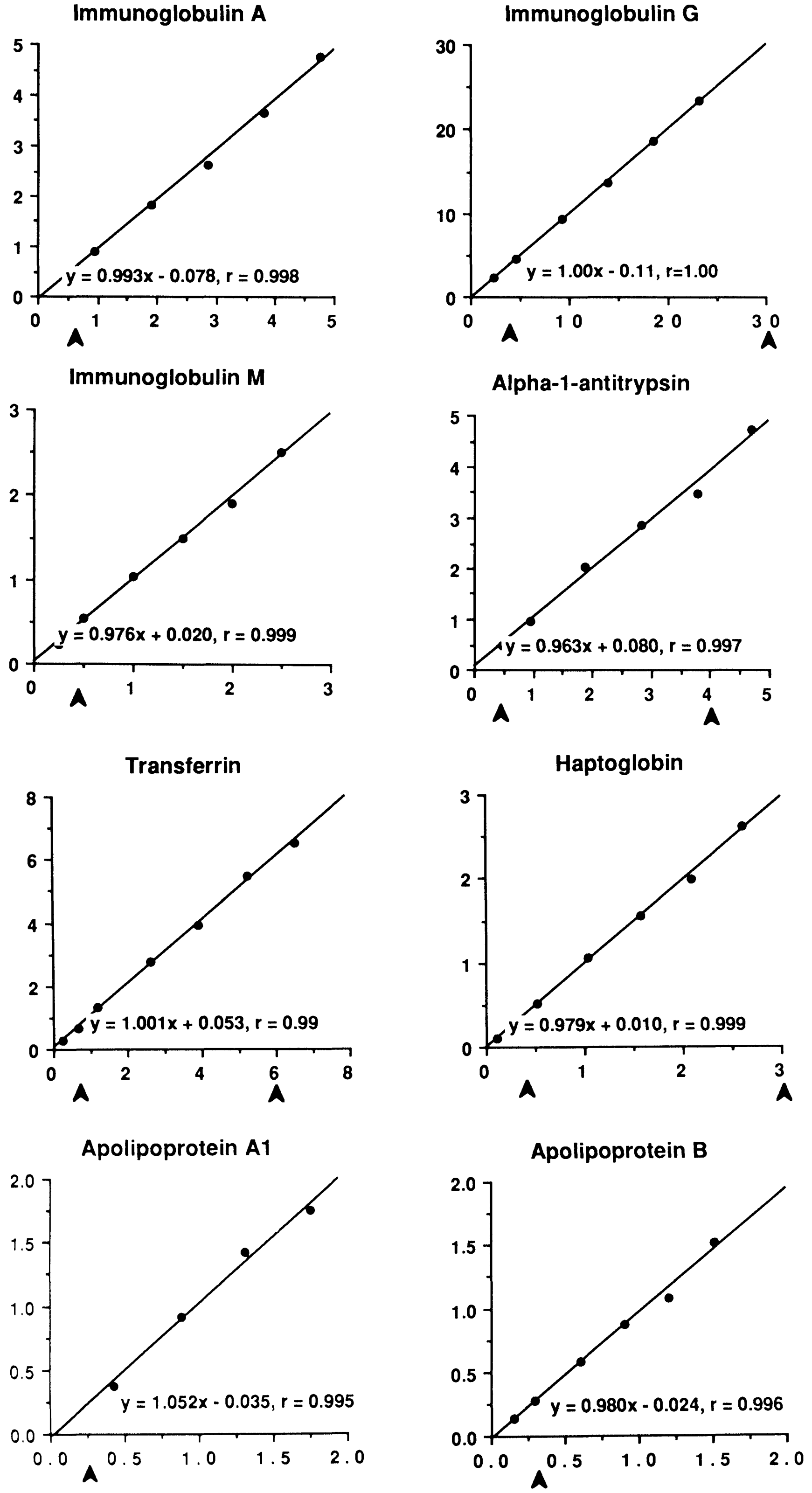

*Figure 2. Linearity. Found concentration (g/l) plotted against calculated concentration (g/l). Working range of primary dilution indicated by arrows.*

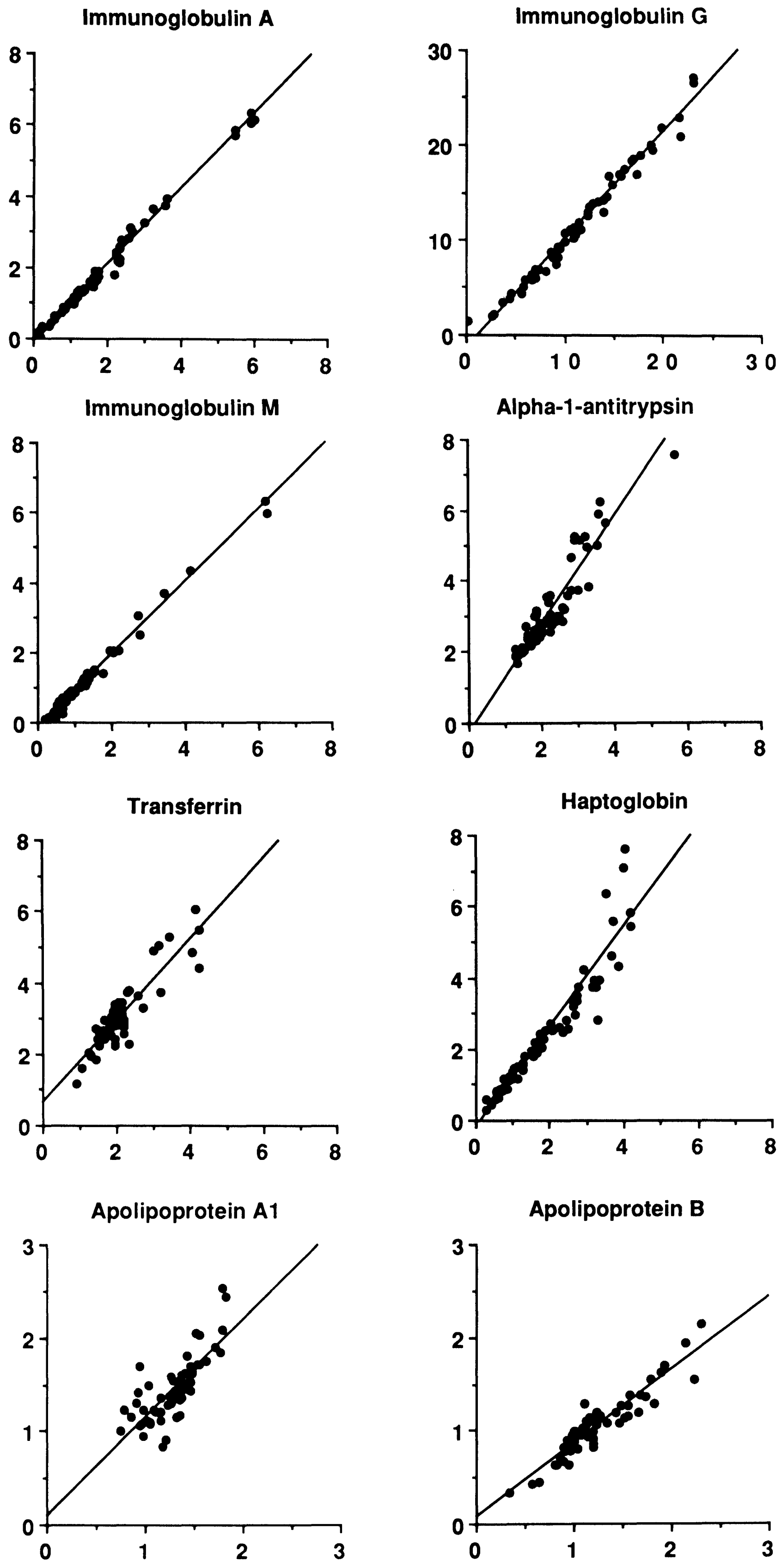

*Figure 3. Comparison studies. Concentration (g/l) measured by DPA*1 plotted against that measured by comparison methodology.*

*Accuracy*

The method comparison studies are shown in figure 3. Good correlation coefficients (table 4) were obtained for all analytes, apart from apolipoprotein A1; particularly good figures were obtained when the comparison method also used a rate nephelometric approach. Slopes significantly different from unity were seen for alpha-1-antitrypsin, haptoglobin and apolipoprotein B, all due to the use of different calibration materials for the comparison method. The concentrations found for a series of commercial calibrators and controls are shown in table 5.

*Table 4. Linear regression statistics for DPA*1 (y-axis) against various comparison methods (x-axis).*

| Test | N | Slope | Y-intercept | Correlation coefficient |
|---|---|---|---|---|
| Immunoglobulin A | 65 | 1·061 | −0·038 | 0·996 |
| Immunoglobulin G | 60 | 1·138 | −1·391 | 0·990 |
| Immunoglobulin M | 64 | 1·036 | −0·145 | 0·990 |
| Alpha-1-antitrypsin | 70 | 1·534 | −0·28 | 0·94 |
| Transferrin | 60 | 1·148 | +0·652 | 0·90 |
| Haptoglobin | 70 | 1·395 | −0·196 | 0·95 |
| Apolipoprotein A1 | 64 | 1·05 | +0·10 | 0·78 |
| Apolipoprotein B | 64 | 0·79 | +0·06 | 0·93 |

The immunoglobulin values obtained for myeloma samples (figure 4[*a*]) were lower than for the comparison method (slope = 0·85), but the concentration measured showed slightly better agreement with results produced by serum electrophoresis (agarose) and densitometric scanning (figure 4[*b*]), whereas agreement between the scanning and comparison method was not as good (data not shown).

*Carry-over*

The sequence $H_1H_2H_3L_1L_2L_3$ was repeated four times for two immunoglobulin G pools and gave the following data:

$L_1$ = 3·796 g/l  SD 0·051

$L_3$ = 3·800 g/l  SD 0·034

H = 28·0 g/l

$L_1$ and $L_3$ are not significantly different, indicating that carry-over was negligible.

*Throughput*

The throughput of the machine is dependent on the number of tests, not samples, and also on whether redilutions and automated antigen excess checking are undertaken. Figure 5 shows the time taken for a variety of workload patterns; thus, 60 tests can be analysed in 30 min if there are no redilutions etc. In the extreme cases using only known myelomas and antigen excess checking on all samples and many redilutions being performed, 100 tests can take approximately 140 min. Overall, a realistic analysis rate appears to be of the order of 100 tests/h.

*Table 5. Comparison of stated concentration and concentration as analysed by DPA*1 for various calibrators and quality assurance materials.*

| Analyte | Material | Stated value† (g/l) | Found value (g/l) |
|---|---|---|---|
| Immunoglobulin A | Atlantic Antibodies CA 1 | 4·86 | 4·36 |
| | Behring N-Protein | 2·38 | 2·15 |
| | Atlantic Antibodies low QC | 0·83 | 0·85 |
| | SPS-01‡ | 2·18 | 2·02 |
| | SPS-02 | 4·83 | 4·44 |
| | EQAS 61 | 1·69 | 1·51 |
| | EQAS 62 | 1·77 | 1·60 |
| | EQAS 63 | 0·38 | 0·32 |
| | EQAS 64 | 42·7 | 53·8 |
| Immunoglobulin G | At AB CA 1 | 26·32 | 24·43 |
| | Behring N-Protein | 11·4 | 10·85 |
| | At AB low QC | 4·32 | 4·23 |
| | SPS-01 | 11·32 | 11·27 |
| | SPS-02 | 25·33 | 23·71 |
| | EQAS 61 | 9·65 | 9·53 |
| | EQAS 62 | 10·41 | 10·20 |
| | EQAS 63 | 2·21 | 1·92 |
| | EQAS 64 | 3·63 | 3·24 |
| Immunoglobulin M | At AB CA 1 | 2·69 | 2·95 |
| | Behring N-Protein | 1·10 | 0·79 |
| | At AB low QC | 0·41 | 0·48 |
| | SPS-01 | 1·21 | 1·16 |
| | SPS-02 | 2·82 | 2·47 |
| | EQAS 61 | 1·37 | 1·76 |
| | EQAS 62 | 1·43 | 1·33 |
| | EQAS 63 | 0·43 | 0·34 |
| | EQAS 64 | 0·28 | 0·16 |
| Alpha-1-antitrypsin | At AB CA 3 | 3·72 | 3·48 |
| | Behring N-Protein | 2·19 | 1·95 |
| | At AB Low QC | 0·77 | 0·64 |
| | SPS-01 | 1·66 | 1·99 |
| | EQAS 61 | 1·44 | 1·49 |
| | EQAS 62 | 1·47 | 1·63 |
| | EQAS 63 | 1·60 | 1·76 |
| | EQAS 64 | 1·14 | 1·13 |
| Transferrin | At AB CA 3 | 5·51 | 5·29 |
| | Behring N-Protein | 3·00 | 3·11 |
| | At AB Low QC | 1·26 | 1·18 |
| | SPS-01 | 2·19 | 3·10 |
| | SPS-02 | 4·87 | 5·9 |
| Haptoglobin | At AB CA 3 | 2·11 | 2·23 |
| | Behring N-Protein | 1·69 | 1·25 |
| | At AB Low QC | 0·54 | 0·59 |
| Apolipoprotein A1 | Immuno Normal QC | 1·27 | 0·82 |
| | Behring Precision L | 1·42 | 1·78 |
| Apolipoprotein B | Immuno Normal QC | 1·00 | 0·52 |
| | Behring Precision L | 0·79 | 0·74 |

† (Or all lab mean for EQAS samples.)
‡ See [3] for details of SPS-01.

*Antigen excess checking*

Table 6 indicates the range of values obtained for the 31 myeloma samples and also whether any antigen excess flags were produced. The figures for IgG indicate the wide range of the assay, with antigen excess not being encountered. Immunoglobulin A produced one failure of the curve fit software in that, for one sample with a low level of IgA, extreme antigen excess was indicated if the 'control add-back' option was used; if only the second sample addition was used the result was correctly recorded as low. In the case of IgM, one myeloma was missed by all three methods of antigen excess checking.

### Discussion

The software, the bar-coded calibrators and the ready-to-use reagents all contributed to the ease of use of the instrument. A level sensor for the PEG diluent would be needed to consolidate the simplicity of use.

Within-day precision was excellent and between-day precision was good and could probably be improved upon in routine use.

Calibration appeared stable throughout a 35 day period in all cases, indicating that monthly calibration should be sufficient. All methods were linear over the assay range, indicating the validity of the curve parameters and single point calibration.

Comparison with other methods was generally good, although the correlation coefficient for apolipoprotein A1 was less good. The significant slopes obtained for some assays is partly due to the difference in assigned values of the relative calibrants, though for alpha-1-antitrypsin and haptoglobin the slope of correlation was greater than the relative concentrations of the Technicon standard in the two measurement systems.

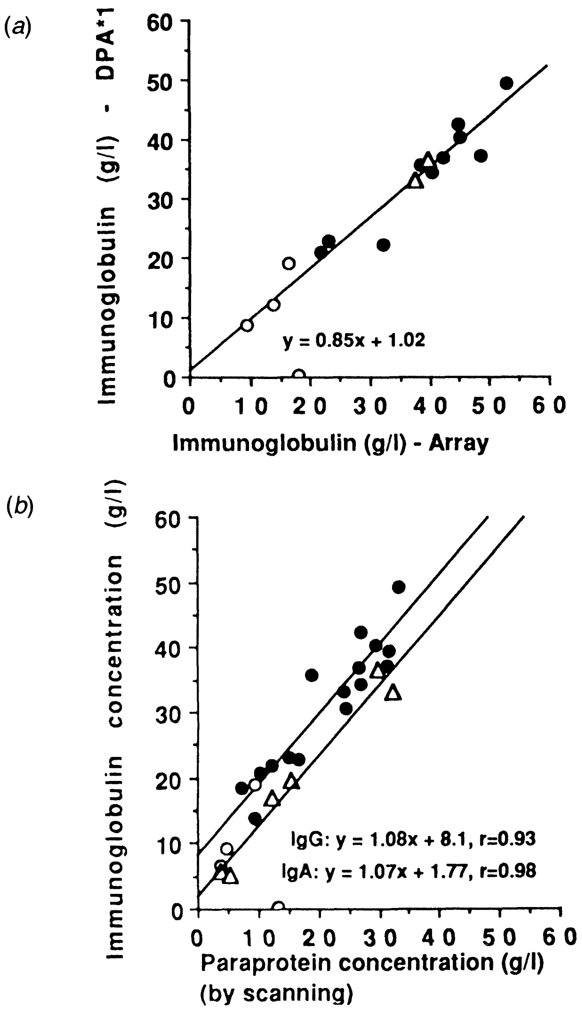

*Figure 4. Comparison studies for samples containing a paraprotein. (a) DPA*1 versus comparison method; (b) DPA*1 versus densitometric scanning,* ● *IgG,* ○ *IgM,* △ *IgA.*

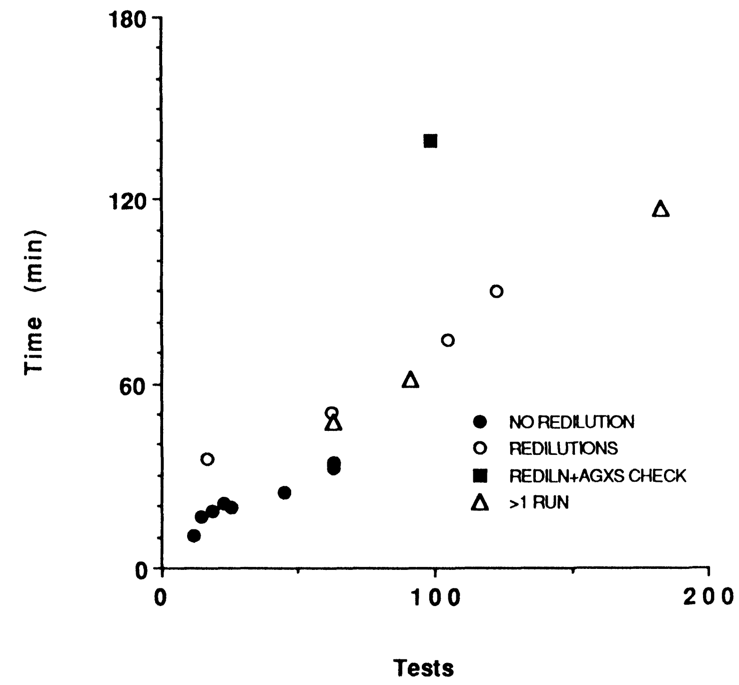

*Figure 5. Evaluation of throughput.*

*Table 6. Results of antigen excess checking software on 31 samples from known myeloma patients.*

| | IgA | IgG | IgM |
|---|---|---|---|
| Number of samples | 7 | 15 | 9 |
| Concentration range | | | |
| Myeloma | 5·1–32·1 | 13·8–49·4 | 6·7–20·8 |
| Overall | 0·06–32·1 | 2·14–49·4 | 0·08–20·8 |
| Number of flags with antigen excess software | 1 (33·1 g/l) | None | 1 Ag Excess<br>1 curve fit failure |
| Number of flags with extreme antigen excess software | 3 correctly flagged low<br>1 extremely Ag XS but result low | None | 1 missed |

The difference in assigned calibration values is reflected in the concentrations measured for different calibrators for the analytes. The DPA*1 is calibrated against the American College of Pathologists' reference preparation, although the software allows for the reassignment of the stated values for the calibrator to be adjusted so as to give results calibrated against a different reference preparation.

The correlation data for transferrin showed a significant intercept on the $y$-axis, although the straight line of the dilution curve indicates that the DPA*1 is probably correct. There was no evidence of carry-over within the system, the washing procedure being satisfactory.

The antigen excess checking procedures are generally satisfactory, although two errors occurred. It is suggested that it is still advisable to run a total protein or serum protein electrophoresis if undertaking immunoglobulin assays for the first time on a patient – this would indicate any samples likely to produce antigen excess in the assay system. For known paraproteins the analyser will give consistent results and such a procedure would be unnecessary [4].

### Acknowledgements

We would like to thank Technicon UK for the loan of the instrument to carry out this work; M. Bubel, Department of Immunology, The London Hospital, for the provision of samples already assayed for immunoglobulins; and Dr J. Calvin, Department of Clinical Biochemistry, Addenbrookes Hospital, Cambridge, UK, for samples with known concentrations of alpha-1-antitrypsin and haptoglobin.

### References

1. Whicher, J. T., Warren, C. and Chambers, R. T., *Annals of Clinical Biochemistry*, **21** (1984), 78.
2. Anderson, R. J. and Sternberg, J. C., A rate nephelometer for immunoprecipitin measurement of specific serum protein. In *Automated Immunoanalysis*, Part II, Ed. Richie, R. F. (Marcel Dekker, New York, 1978), 409.
3. Milford Ward, A., White, P. A. E. and Thompson, R. A. *et al.*, *Annals of Clinical Biochemistry*, **21** (1984), 254.
4. Whicher, J. T., Calvin, J., Riches, P. and Warren, C., *Annals of Clinical Biochemistry*, **24** (1987), 119.

